# Lower bone mineral density in Somali women living in Sweden compared with African–Americans

**DOI:** 10.1007/s11657-015-0208-5

**Published:** 2015-02-19

**Authors:** Taye Demeke, Gamal Abd El-Gawad, Amra Osmancevic, Martin Gillstedt, Kerstin Landin-Wilhelmsen

**Affiliations:** 1 Hjällbo Primary Health care, S-424 32 Gothenburg, Sweden; 2 Family Health Primary Health Care, Gothenburg, Sweden; 3 Department of Dermatology, Sahlgrenska University Hospital, Gothenburg, Sweden; 4 Section for Endocrinology, Institution of Medicine, Sahlgrenska University Hospital at Sahlgrenska Academy, University of Gothenburg, Gothenburg, Sweden

**Keywords:** Bone mineral density, Vitamin D, African–American, Immigrants

## Abstract

***Summary*:**

Vitamin D deficiency can lead to osteomalacia. Bone mineral density was lower in Somali women, living in Sweden, in relation to both the American and the African–American reference populations. The majority, 73 %, had vitamin D deficiency, and supplementation should be considered to prevent from osteomalacia, osteoporosis and future fractures.

**Purpose:**

Low vitamin D can lead to osteomalacia. The hypothesis was that bone mineral density (BMD) in Somali women living in Sweden was lower in comparison with different ethnic reference populations.

**Methods:**

Women from Somalia, *n* = 67, median age 35.8 years (range 18 to 56), latitude 0–10° North living in Gothenburg, Sweden, latitude 57° North, >2 years were studied. All wore traditional Islamic clothing and had skin photo type V. BMD was recorded as the *Z*-score and compared with white American and African–American women, respectively, using standard data from the dual energy X-ray absorptiometry (DXA) manufacturer (Lunar Prodigy enCORETM, GE Healthcare, LU44663). A fasting blood test was drawn for analysis of serum 25(OH)D.

**Results:**

The median *Z*-score compared with the American white population was −0.9 SD of the lumbar spine (*p* < 0.00001), 0.1 SD of the left hip and 0.0 SD of the right hip (ns).

The median *Z*-score compared with the African–American population was −1.6 SD of the lumbar spine (*p* < 0.00001), −0.9 SD of the left hip and −0.9 SD of the right hip (*p* < 0.001). The majority, 73 %, had vitamin D deficiency, serum 25(OH)D <25 nmol/l (<10 ng/ml). BMD did not correlate to vitamin D levels or to the number of years in Sweden. One wrist fracture was reported.

**Conclusions:**

BMD was lower in these fairly young immigrant women from Somalia, living in Sweden, in relation to both the American and the African–American reference populations. Vitamin D supplementation should be considered to prevent from osteomalacia, osteoporosis and future fractures.

## Introduction

Vitamin D deficiency is considered to be an epidemic of worldwide proportion, involving all races and all age groups [1]. It is an important determinant of skeletal development and maintenance of bone mass throughout life [2]. Vitamin D promotes calcium absorption and bone mineralisation and is positively associated with bone mineral density (BMD) [3]. The level of vitamin D required to attain optimal bone health, however, is still unclear [4]. The existence of ethnic variations in BMD is recognized [5], but the underlying mechanism accounting for the differences is not yet fully understood. It is well established that African–Americans have lower fracture risk due to higher BMD compared with their white counterparts in the USA [6]. Somali women and Sudanese immigrants in the USA [7], despite sharing the same African heritage, exhibit low BMD which in part was ascribed to malnutrition in sub-Saharan Africa [8]. It appears that there exists a specific bone metabolic mechanism favouring blacks of the West African origin [9].

The negative health effects of vitamin D deficiency and low BMD in the immigrant minority population are not yet studied in Sweden. Thus, the purpose was to study BMD status of Somali women living in Sweden compared to African–American women and white American women in the USA using data supplied by the dual energy X-ray absorptiometry (DXA) manufacturer. Serum vitamin D levels were also measured. The aim was to test the hypothesis that BMD in Somali women living in Sweden was lower than that in the white and African–American reference population.

## Methods

A total number of 67 Somali women from latitude 0–10° N, age 18–56 years, living in Gothenburg, Sweden, latitude 57° N, since at least 2 years, were recruited on a voluntary basis via flyers to Somali community shops and placed advertisements at healthcare units, a nearby pharmacy and a super market. It is estimated to be around 4000 Somalis in Gothenburg with a total population of 500,000. Different Somali associations were contacted to inform about the objective of the study. All subjects willing to participate, *n* = 104, were referred to our research assistant and were examined by an experienced physician (TD) before enrolment. DXA was performed in 67 women and 37 were lost to follow-up. All but 1 was premenopausal.

Weight (kg) was measured wearing indoor clothing without shoes. Height (cm) was measured using wall-mounted stadiometer without shoes. Body mass index (BMI) was calculated as a body weight divided by the height squared (kg/m^2^).

BMD measurements were made by DXA at the lumbar spine and the left and right hip. The coefficients of variation for GE/LUNAR device at the lumbar and femoral neck were 1.22 and 1.97. BMD was recorded as the *Z*-score (difference in SD from the mean of healthy, matched women). These results were adjusted for age, body weight, gender and skin type for both patients and referents. A *Z*-score of −2.0 or less signifies low BMD for age, www.ISCD.org. The BMD in Somali women was compared both with BMD in white American women and BMD in African–American women using standard data provided by the DXA manufacturer (LUNAR Prodigy enCORETM, GE Healthcare, LU44663). A fasting blood test was drawn for analysis of serum 25(OH)D (DiaSorin, Stillwater, MN, USA) and serum intact parathyroid hormone (S-PTH) (ABBOT, Architect, Wiesbaden, Germany). Photometry 600 nm was used to determine concentrations of S-calcium and bone-specific alkaline phosphatase (ALP). Ionized calcium in serum was measured using ion-selective electrodes.

Participants completed a questionnaire regarding recent medication both prescribed and none prescribed, parity, duration of residency in Sweden, smoking habits, previous diseases and fractures. Subjects also filled in a visual analogue scale (VAS), 0–10 cm (low to high), in order to evaluate pain. Quality of life, including physical and mental scores, was self-recorded by Short Form (SF) −36, 0–100 (low to high), with questionnaires [10]. The survey was conducted in Gothenburg, during autumn and winter 2010, 2011 and 2012. Eligibility criteria for inclusion comprised no history of serious disease states, no medication that might interfere or affect bone metabolism, no pregnancies and no lactations.

A written consent was obtained, and the project was approved by the Swedish research ethical committee at the University of Gothenburg.

### Statistical analyses

Mean, standard deviations and medians were calculated with conventional methods. Wilcoxon’s signed rank test was used to test for differences between subject BMD values and the reference distribution. Correlations were performed with Spearman’s test. *p* < 0.05 was considered statistically significant.

## Results

**Fig. 1:**
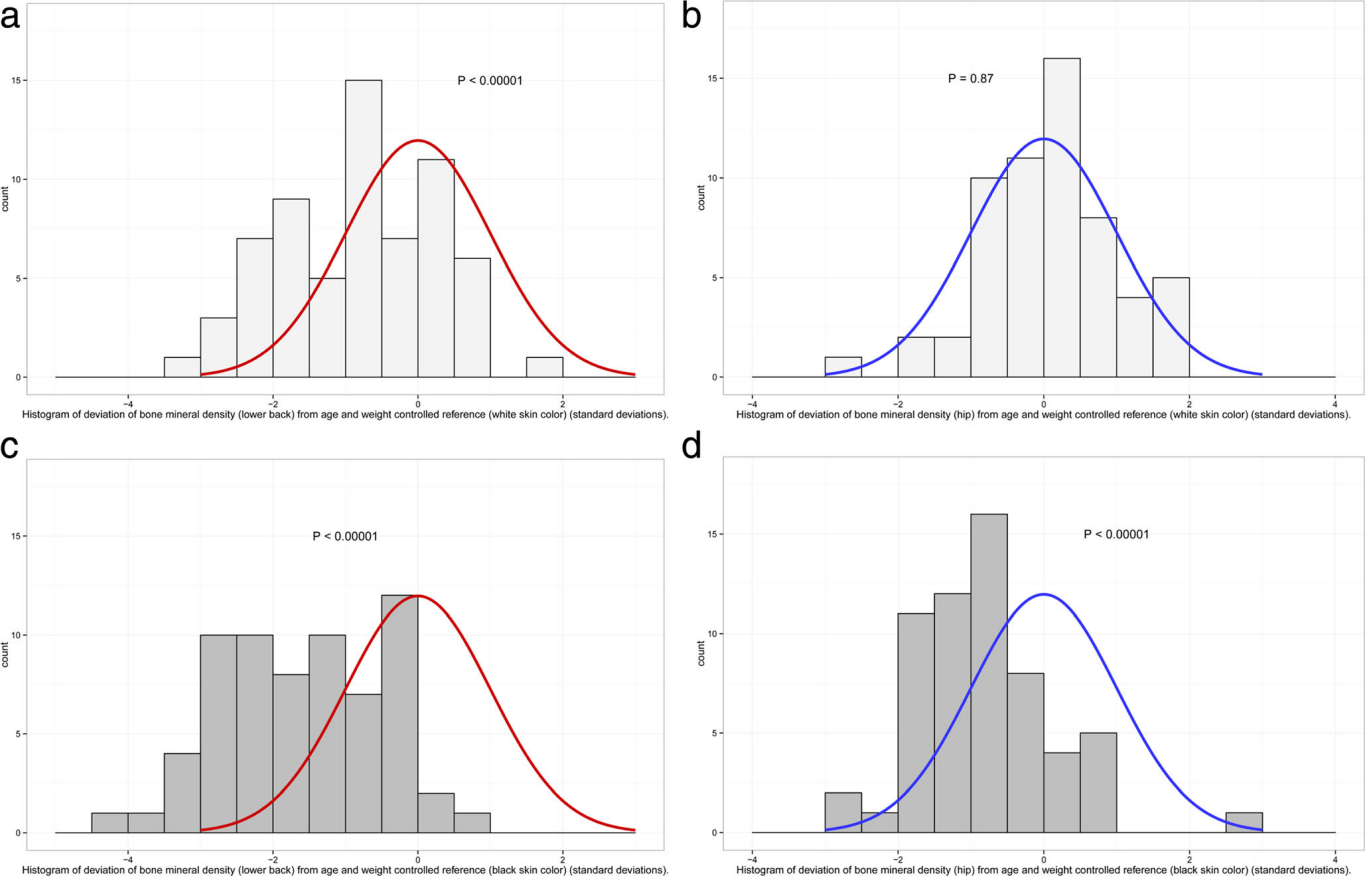
**a** to **d** Histogram of bone mineral density (BMD) as *Z*-score (SD from the reference mean) in 67 Somali, premenopausal women compared with white American, lumbar spine (**a**), mean of right and left hip (**b**), and compared with African–American women, lumbar spine (**c**), mean of right and left hip (**d**), according to LUNAR dual energy X-ray absorptiometry (DXA) device reference populations. Count = number of subjects in each bin. The *curves* indicate the normal distribution according to the manufacturer’s reference population

Anthropometric and background data, VAS, SF-36 scoring and BMD data are given in Table 1. The median value of *Z*-score compared with the reference interval for the American white population was −0.9 SD (min −3.4, max 1.6) of the lumbar spine (*p* < 0.00001), 0.1 SD (min −1.7, max 2.1) of the left hip (ns) and 0.0 SD (min −2.0, max 1.7) of the right hip (ns) (Fig. 1a, b).

**Table 1:** Characteristics of the 67 Somali women who performed bone mineral density measurement with dual energy X-ray absorptiometry (DXA). Mean ± SD and range are given for continuous variables

	Mean ± SD	Range Min–max	Number of patients with data
Age, years	35.1 ± 9.2	18–55.9	67
Height, cm	163.6 ± 6.9	151–182	67
Body weight, kg	72.1 ± 13.7	39–99	67
BMI, kg/m^2^	27.0 ± 5.1	16.9–37.3	67
Years in Sweden	14.5 ± 6.2	3–24	65
Parity, *n*	2.8 ± 2.6	0–11	64
Non-smokers, *n* (%)	0 (100 %)		67
Fractures, *n*	1	0–1	67
S-25(OH)D, nmol/l	22.4 ± 13.0	0–72	67
S-25(OH)D <25 nmol/l = deficiency, *n* (%)	49 (73.1 %)		67
S-calcium, mmol/l	2.32 ± 0.09	2.15–2.26	67
S-ionized calcium, mmol/l	1.23 ± 0.04	1.17–1.21	67
S-bone-specific ALP, μkat/l	14.7 ± 5.6	6.0–34.0	67
S-PTH, pmol/l	5.60 ± 1.98	1.81–11.20	67
VAS, pain score, low to high 0–10 cm	6.6 ± 2.7	1–10	37
Physical capacity score, low to high 0–100	41.5 ± 12.1	9–63	45
Mental capacity score, low to high 0–100	37.2 ± 13.3	3–62	45
Bone mineral density, lumbar spine, g	1065 ± 120	778–1367	67
Bone mineral density, right hip, g	961 ± 110	688–1222	59
Bone mineral density, left hip, g	968 ± 119	705–1236	59
Bone mineral density, mean hip, g	970 ± 110	705–1222	61

*S-ALP* serum alkaline phosphatase, *S-PTH* serum intact parathyroid hormone, *VAS* visual analogue scale

The median value of *Z*-score compared with reference interval for the African–American population was −1.6 SD (min −4.1, max 0.9) of the lumbar spine (*p* < 0.00001), −0.9 SD (min −2.8, max 1.0) of the left hip (*p* < 0.00001) and −0.9 SD (min −3.0, max 2.2) of the right hip (*p* < 0.00001) (Fig. 1c, d).

Eleven (16 %) and 26 (39 %) women had low BMD for age according to the American and African–American referents, respectively. The oldest woman, 56 years, had osteoporosis and reported a wrist fracture.

Vitamin D deficiency, serum 25(OH)D <25 nmol/l (<10 ng/ml) was found in 73 %, and only 4 women (6 %) had 25(OH)D >50 nmol/l (Table 1). Mean S-PTH was 5.60 ± 1.98, median 5.18 pmol/l and reference levels 1.60–6.90 pmol/l. Mean S-calcium was 2.32 ± 0.09, median 2.32 mmol/l, reference levels 2.15–2.50 mmol/l; mean serum ionized calcium was 1.23 ± 0.04, median 1.23 mmol/l, reference levels 1.18–1.31 mmol/l; and mean serum bone-specific ALP was 14.7 ± 5.6, median 13.5 μg/l, reference values 2.9–14.3 μg/l (Table 1).

There was no correlation between BMD at any region of interest and age, serum 25(OH)D, number of years in Sweden, parity, pain according to VAS or physical or mental capacity score according to SF-36, respectively.

## Discussion

BMD was lower in the present Somali women than in the African–American referents for LUNAR DXA. BMD of the lumbar spine was also lower than in the white American referents. Vitamin D deficiency was frequent. However, no relation between BMD and duration of stay in Sweden or in relation to vitamin D levels was observed. Our results confirm a previous study with lower BMD of the forearm in a group of immigrant women in Finland at similar latitude [11]. The present results show that BMD was lower also at weight-bearing regions at the lumbar spine and hips. Fractures were rare in these fairly young women.

The Somali women of the present study scored pain of a fairly high degree, but there was no relation between BMD and reported pain according to VAS. Poor muscle strength, vague musculoskeletal pain and low BMD are some of the clinical manifestations of hypovitaminosis D [12]. DXA application to detect osteomalacia is inconclusive, and a bone biopsy may be mandatory for histological diagnosis [13]. At higher ages, after the menopause, more women might precipitate osteoporosis, i.e. more BMD loss, during DXA measurement. Somali dietary habits, with less frequent intake of food containing vitamin D and calcium, wearing long robes and dresses, avoidance of sun exposure [14], having dark skin and living at northern latitudes [15] might contribute to the vitamin D deficiency.

There is a strong evidence showing that vitamin D plays a vital role in calcium metabolism, leading to bone ossification and bone remodelling and, hence, to maintain the BMD [1]. The present Somali women had lower spinal BMD in comparison to white women. This is in concordance with a study in the USA involving Somali immigrant women, African–American women and white women, which showed higher spinal BMD value in African–American women than in both Somali and white women [9]. Somali immigrants in the USA and Somalis living in Finland did not exhibit the same robust BMD value compared with African–American women despite sharing the same African ancestry [11]. The possibility of genetic influences on BMD favouring the African–American group has also been discussed [16].

Low BMD defined as osteoporosis is a strong predictor for fractures [17]. Low BMD for age was found in 39 % of the fairly young and premenopausal Somali women of this study. In Sweden, osteoporosis-linked fractures and morbidity incur a substantial medical expenditure and loss of quality of life [18].

The prevalence of osteoporosis in minority population is not yet explored in Sweden. Somalis are among the fastest growing groups of foreign-born residents in Sweden. Increased morbidity related to osteoporosis and fracture might incur a significant strain on the healthcare system in Sweden in the future when these women reach the postmenopausal stage and the fracture ages.

No correlation could be seen between BMD and duration of stay in Sweden. However, a larger sample might be needed to reach a significant correlation. The mean duration of years in Sweden was 14 years which must be considered as long. Other factors as physical inactivity might be an even stronger predictor for BMD in this group of women. The degree of physical activity in native Swedish women correlated positively with S-25(OH)D, independently of other factors, in a random population sample of similar age in Gothenburg, as the present Somali women [19]. Physical activity is connected with being outdoors and thereby an increased sun exposure. There are reports of a lower degree of physical activity and staying indoors to a large extent in immigrant women at higher latitudes [15, 20] and could to some extent explain the lower BMD in Somali women living in Sweden.

Hypovitaminosis D was extensive in this study group, with 73 % having deficiency. The majority also had elevated S-PTH or in the upper reference interval, and serum bone-specific ALP levels were high. This suggests the need for supplementation to avoid demineralisation and further BMD loss and fractures. Encouraging the use of vitamin D supplements and following physician recommendations are emphasized. Information is important, and it was shown that immigrants with longer stay in their new country had a better awareness of osteoporosis and prevention [20].

One limitation was that bone biopsy was not performed for a definite diagnosis of osteomalacia. The procedure can be cumbersome and uncomfortable for the participants and difficult to motivate, making recruitment endeavours of such large group unattainable. A higher number of participants would probably be necessary to get statistical power for BMD correlations with duration of stay in Sweden. Age-matched native controls from Sweden had been of value. However, no comparisons could then have been performed with subjects of similar skin photo type. The LUNAR’s African–American DXA reference population was based on BMD measures of women from Egypt according to the manufacturer.

A study strength was the engaging of a “hard to reach” group in research. This is often very difficult. Against all odds, we were able to recruit a fairly large number of premenopausal Somali women. It is also important to have only one national entity as genetic differences are found regarding BMD depending on the origin. BMD measurements were performed at both regions of interest, lumbar spine and hips and not limited to forearm, unlike other studies. An attempt was also made to evaluate the BMD relation to duration in Sweden, to parity and to degree of pain.

In conclusion, lumbar spine BMD in Somali women living in Sweden was lower than that in the white American DXA reference population. BMD on all regions of interest were lower than the African–American DXA referents. Vitamin D deficiency was common, and supplementation is advised to prevent from osteomalacia, osteoporosis and future fractures.
